# A systematic review of mortality and long-term functional outcomes after decompressive craniectomy for acquired brain injury: The missing rehabilitation reporting

**DOI:** 10.3934/Neuroscience.2026023

**Published:** 2026-09-09

**Authors:** Davide Giannuzzi, Claudia Lombardo, Pierluigi Contaldi, Maria Caputo, Francesco Pambianco, Gianpaolo Ronconi, Francesco Signorelli, Francesco Doglietto, Augusto Fusco

**Affiliations:** 1 High-Intensity Neurorehabilitation, Department of Neuroscience, Sensory Organs and Thorax, Fondazione Policlinico Universitario “A. Gemelli” IRCCS, Rome, Italy; 2 Department of Geriatrics and Orthopaedics, Catholic University of the Sacred Heart, Rome, Italy; 3 Department of Neurology, Carpi Hospital, 41012 Modena, Italy; 4 Department of Biomedical, Metabolic and Neural Sciences, University of Modena and Reggio Emilia, 41125 Modena, Italy; 5 Department of Neuroscience, Catholic University of the Sacred Heart, Rome, Italy; 6 Physical and Rehabilitation Medicine, Department of Ageing, Orthopaedic and Rheumatological Sciences, Fondazione Policlinico Universitario “A. Gemelli” IRCCS, Rome, Italy; 7 Neurosurgery, Department of Neuroscience, Sensory Organs and Thorax, Fondazione Policlinico Universitario “A. Gemelli” IRCCS, Rome, Italy

**Keywords:** decompressive craniectomy, outcome, functional outcome, rehabilitation scales, acquired brain injury

## Abstract

**Background:**

Decompressive craniectomy (DC) is a surgical procedure for refractory intracranial hypertension following severe acquired brain injury (sABI) and is an effective, life-saving treatment. However, there is significant controversy regarding its impact on long-term functional outcome. Furthermore, the role of postoperative rehabilitation in shaping recovery after DC has been largely overlooked in the literature.

**Objectives:**

To systematically review randomized controlled trials (RCTs) evaluating the long-term functional outcomes of DC in patients with sABI, stratified by underlying etiology, with particular attention to the assessment tools employed, their alignment with the International Classification of Functioning, Disability and Health (ICF) framework, and the reporting of post-operative rehabilitation data.

**Methods:**

We searched PubMed and Scopus for all RCTs related to DC published between 2000 and 2024. Eligible studies included adults (≥18 years) who underwent DC for acute brain injury and reported at least one functional outcome measure (GOS-E, mRS, GOS, or Barthel Index). Outcomes were dichotomized into favorable and unfavorable categories. A narrative summary was performed because of the substantial clinical and methodological heterogeneity.

**Results:**

We included 16 RCTs (1803 participants) in our analysis. The etiologies included traumatic brain injury, intracerebral hemorrhage, acute subdural hematoma, and malignant cerebral infarction. DC was associated with lower overall mortality compared to those not receiving it (24% vs. 31%). However, in 8 RCTS, a higher proportion of patients with DC had unfavorable functional outcomes. All outcome scales mapped exclusively to the ICF domain of “Activities and Participation”, while any assessment of cognitive function, emotional well-being, social participation, or quality of life was reported. Rehabilitation was largely unmeasured rather than necessarily absent: Only one study reported any rehabilitation data, limited to the number of patients admitted to inpatient rehabilitation. Most RCTs reported outcomes at 6 months or before; only one RCT extended the follow-up period to 24 months.

**Conclusions:**

DC is an effective, life-saving procedure that reduces mortality and has an etiology-dependent effect on functional outcomes. Available functional outcome measures do not adequately capture the multifaceted nature of recovery following severe brain injury. Crucially, the post-DC rehabilitation trajectory is not captured by current trials; this argues for the routine, prospective collection of rehabilitation data in future RCTs to determine the contribution of postoperative rehabilitation to long-term functional recovery rather than against the use of a life-saving procedure.

## 1. Introduction

Decompressive craniectomy (DC) is a life-saving surgical procedure designed to mitigate dangerously elevated intracranial pressure (ICP) by removing a portion of the skull [1]. DC is frequently employed in patients with elevated ICP resulting from etiologies refractory to less invasive treatments such as traumatic brain injury (TBI) and ischemic stroke [2]. Although several studies and randomized controlled trials (RCTs) have demonstrated the efficacy of this procedure in reducing ICP and mortality [3],[4], there is controversy regarding its effectiveness, with studies showing contrasting results in terms of long-term disability [5]. Furthermore, the impact of DC on quality of life remains inadequately investigated.

This discrepancy may be attributable to several factors. The first is methodological differences between studies that complicate comparisons of results. Methodological differences across studies complicate comparisons of results, including variations in patient populations (e.g., patient selection criteria), timing of surgery (e.g., early versus late in the disease course), and pre-injury neurological function. Furthermore, specific factors contribute to better or worse outcomes in patients with disorders of consciousness, particularly those who are younger and with higher Glasgow Coma Scale (GCS) scores after the injury [6].

Second, most studies on DC have focused on traumatic brain injuries. However, there is a relative paucity of research and older literature on DC for other etiologies [7]–[9].

Third, there is a notable lack of information regarding the recovery process following the procedure. Studies have not consistently described the type, intensity, or duration of postoperative neurorehabilitation, making it difficult to estimate the specific contribution of DC to the functional outcomes. The use of different scales to evaluate disability may also contribute to this lack of clarity [10]. Most researchers evaluating long-term functional outcomes after DC rely on scales such as the modified Rankin Scale (mRS), Glasgow Outcome Scale (GOS), or Glasgow Outcome Scale Extended (GOS-E). These instruments inadequately capture crucial dimensions of overall recovery, including cognitive, emotional, and quality-of-life outcomes, and may therefore underestimate the residual disability. Furthermore, domains such as communication, executive functioning, and social reintegration, alongside contextual factors, including environmental and personal variables, are largely absent from the evaluation of functional outcomes after DC. Integrating assessment tools capable of capturing these broader life domains would address a major gap in the literature and align outcome measurements with patient-centered care [11].

Finally, outcomes are commonly measured at 6 months, with few researchers extending follow-up beyond 12 or 24 months [12]. It should be noted that the effectiveness of a treatment for severe brain injury requires longer assessment periods, as symptoms of TBI may persist for decades, affecting cognitive capabilities, psychosocial functioning, and quality of life.

Our purpose of this study was to conduct a systematic review of RCTs to determine the impact of DC on long-term outcomes in patients with severe brain injury. We also considered the surgical and clinical characteristics of patients who underwent DC, with particular attention to rehabilitative parameters, including intensity and type. Because the natural history and treatment effect of DC differ substantially across etiologies, findings are interpreted stratified by underlying etiology rather than as a single uniform intervention, and the long-term functional and rehabilitative trajectory of survivors is specifically analysed.

## 2. Materials and methods

### 2.1. Search strategy

This systematic review was conducted in accordance with the Preferred Reporting Items for Systematic Reviews and Meta-Analyses (PRISMA) guidelines [13] (Figure 1).

**Figure 1.**
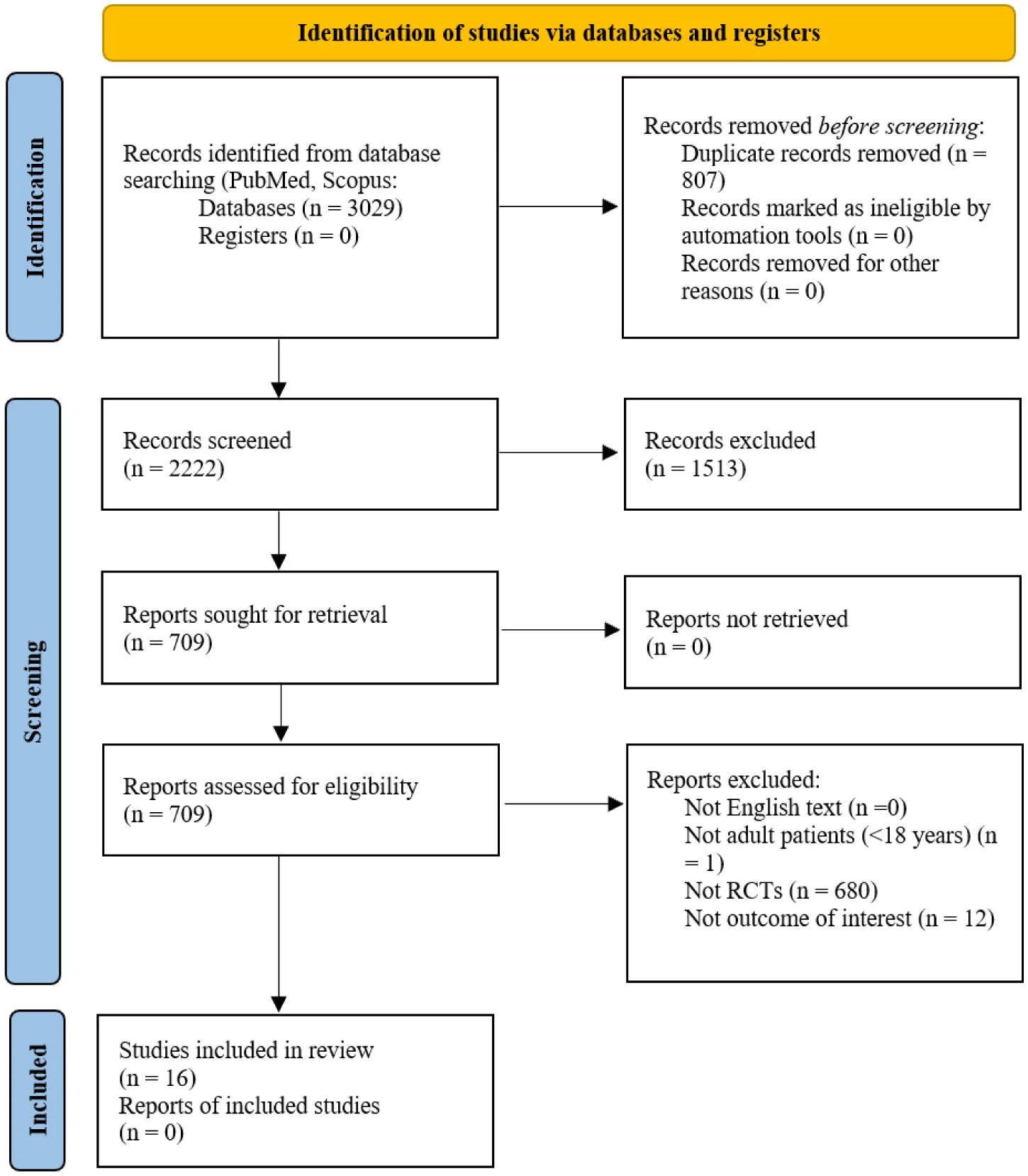
PRISMA 2020 flow diagram for new systematic reviews.

A systematic literature search, limited to RCTs, was conducted for PubMed and Scopus for articles published between 2000 and 2024. The search strategy is presented in Table 1. The string deliberately combined the intervention term (decompressive craniectomy) with rehabilitation- and outcome-related descriptors (e.g., Rehabilitation, Critical Care Outcomes, Patient Outcome Assessment, Fatal Outcome) to maximize the retrieval of trials reporting functional and rehabilitation outcomes, which were our focus of this review. The study design was not restricted at the level of the search string; instead, the RCT requirement was applied during screening against the eligibility criteria described in Section 2.2, so that the search and eligibility criteria remained aligned.

**Table 1.** Keyword string search for each database.

Keywords
Pubmed ((Rehabilitation[MeSH Terms]) OR (Stroke Rehabilitation[MeSH Terms]) OR (Critical Care Outcomes[MeSH Terms]) OR (Physical and Rehabilitation Medicine[MeSH Terms]) OR (Treatment Outcome[MeSH Terms]) OR (Patient Outcome Assessment[MeSH Terms]) OR (Fatal Outcome[MeSH Terms]) AND (Decompressive Craniectomy[MeSH Terms])) AND (Decompressive Craniectomy[MeSH Terms])
Scopus (TITLE-ABS-KEY(rehabilitation) OR TITLE-ABS-KEY(“stroke rehabilitation”) OR TITLE-ABS-KEY(“critical care outcomes”) OR TITLE-ABS-KEY(“physical and rehabilitation medicine”) OR TITLE-ABS-KEY(“treatment outcome”) OR TITLE-ABS-KEY(“patient outcome assessment”) OR TITLE-ABS-KEY(“fatal outcome”)) AND TITLE-ABS-KEY(“decompressive craniectomy”)

### 2.2. Eligibility criteria

We evaluated RCTs that assessed functional outcomes in adults (≥18 years) who had previously undergone DC secondary to brain injury. Included studies measured at least one of the following scales to assess function, administered before and after the procedure: the GOS-E, mRS, or Barthel index (BI). Studies comparing DC with alternative surgical techniques or standard medical therapies were also included.

Studies were excluded if they did not report at least one of the aforementioned assessments at either time point. Non-English reports, case reports, short reports, conference abstracts and posters, and systematic reviews were also excluded.

### 2.3. Study selection

Following duplicate removal, two authors independently screened the titles and abstracts to identify potentially eligible studies. Subsequently, the same reviewers evaluated the full texts of all the identified studies to determine their inclusion status. When disagreements arose regarding a study's inclusion status, the reviewers resolved them through consensus discussions; otherwise, a third reviewer assisted in resolving the disagreement.

### 2.4. Data extraction

Two reviewers independently extracted the data using a predefined spreadsheet. The following information was collected: number of participants, sex distribution, mean age, etiology of brain injury, type of DC and comparator, mortality rate, follow-up duration, rehabilitation setting (as reported), and functional outcome measures.

### 2.5. Data synthesis

Functional outcomes were dichotomized into favorable and unfavorable categories, where feasible. In the primary analysis, unfavorable outcomes were defined as GOS 1–3, GOS-E 1–4, mRS 5–6, or BI ≤ 35, whereas favorable outcomes were defined as GOS 4–5, GOS-E 5–8, mRS 0–4, or BI ≥ 35. When individual studies utilized different cutoffs, their categories were reclassified to align with these thresholds to the extent that the data allowed; otherwise, the authors' original definitions were reported. Reclassification was performed using aggregate (group-level) data published in each report, as individual patient data were not available. This was feasible only when the published score distribution mapped unambiguously onto the predefined thresholds; when a study reported outcomes using a single, non-overlapping cut-off that could not be re-expressed at the patient level, the original dichotomy was retained and flagged as such. Therefore, this approach is approximate, and residual misclassification at the boundary between categories cannot be excluded; the corresponding uncertainty is acknowledged as a limitation.

Owing to many differences among studies regarding the clinical or methodological issues that affected how each study was conducted (etiology, surgical technique, comparison group, and/or definition of outcomes), a quantitative meta-analytic approach was considered inappropriate. Therefore, the results are provided in narrative and tabular formats for descriptive purposes.

Each functional outcome measure used in this review was compared with the ICF categories of function. Furthermore, where possible, information related to postoperative rehabilitation, including the location, timing, frequency, and/or type of treatment, was collected. Given that the clinical question, natural history, and expected treatment effect of DC differ markedly across pathologies, the synthesis was structured by etiology (traumatic brain injury, malignant middle cerebral artery infarction, intracerebral hemorrhage, and acute subdural hematoma) rather than pooling all conditions together, and the direction of effect on mortality and functional outcomes was summarized separately for each etiology.

### 2.6. Risk of bias assessment

The methodological quality of all the included studies was evaluated using the Cochrane Risk of Bias tool for randomized trials, version 2 (RoB 2) [14]. This tool assesses the risk of bias across five domains: (1) Bias arising from the randomization process, (2) bias due to deviations from intended interventions, (3) bias due to missing outcome data, (4) bias in the measurement of the outcome, and (5) bias in the selection of the reported result. For each domain, the risk of bias was judged as “low”, “some concerns”, or “high” (Figure 2).

**Figure 2.**
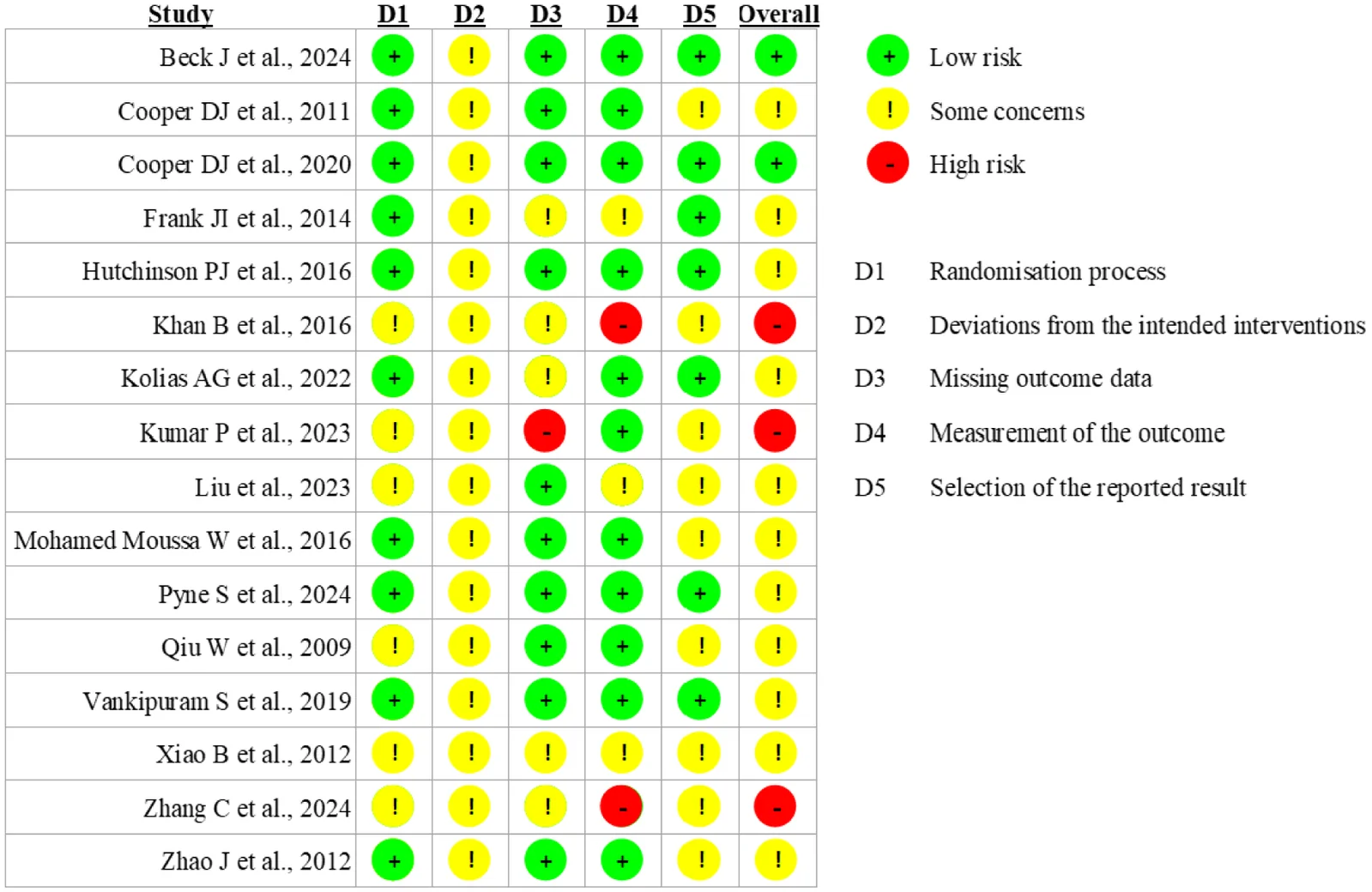
Risk of bias in the included studies.

## 3. Results

### 3.1. Study selection and patient characteristics

Sixteen RCTs published between 2000 and 2024 and meeting the selection criteria (Table 2) evaluated functional outcomes in patients with a variety of acute brain injuries. There were also two publications [12],[15] on longer-term follow-up information from two studies that had been previously included in this review [3],[4]; therefore, the 16 items were related to 14 independent cohorts.

**Table 2.** Study characteristics.

**Authors, year**	**Population**		**Etiology**	**Control Group**	**Mortality (DC vs C)**	**Follow-up (Months)**
	**N (DC vs. C)**	**Age (years, mean DC vs. C)**				
Beck J et al., 2024 [16]	197 (96 vs. 101)	60 vs. 61	IH	Medical Group	17%–22% vs. 27%–31%	6–12
Cooper DJ et al., 2011 [3]	155 (73 vs. 82)	23.7 vs. 24.6	TBI	Medical Group	19% vs. 18%	6
Cooper DJ et al., 2020 [15]	155 (73 vs. 82)	23.7 vs. 24.6	TBI	Medical Group	21% vs. 19%	12
Frank JI et al., 2014 [17]	24 (14 vs. 10)	52.3 vs. 57.9	LSCHI	Medical Group	36% vs. 40%	3– 6
Hutchinson PJ et al., 2016 [4]	398 (202 vs. 196)	32.3 vs. 34.8	TBI	Medical Group	26.9%–30.4% vs. 48.9%–52%	6–12
Khan B et al., 2016 [18]	59 (31 vs. 28)	33.4	ASDH	Craniectomy With Multidural-Slits	45.16% vs. 53.57%	1,5
Kolias AG et al., 2022 [12]	398 (202 vs. 196)	32.3 vs. 34.8	TBI	Medical Group	33.5% vs. 54%	24
Kumar P et al., 2023 [19]	120 (60 vs. 60)	36.65 vs. 37.28	TBI	DC Without Dural Closure	10% vs. 8%	3
Liu C et al., 2023 [20]	86 (43 vs. 43)	50 vs. 51	TBI	Stepwise Intracranial Decompression (SID)	11.63% vs. 2.33%	6
Moussa WM et al., 2017 [21]	40 (20 vs. 20)	59.3	IH	Hematoma Evacuation	10% vs. 25%	3–6
Pyne S et al., 2024 [22]	248 (122 vs. 126)	51.7 vs. 52.3	ASDH	Craniotomy	nd	12
Qiu W et al., 2009 [23]	74 (37 vs. 37)	39.9 vs. 40	TBI	Temporoparietal Craniectomy	27% vs. 57%	1
Vankipuram S et al., 2020 [24]	115 (59 vs. 56)	37.5 vs. 40.2	TBI	Four-Quadrant Osteoplastic Decompressive Craniotomy (Foqosd)	37.9% vs. 45.4%	6
Xiao B et al., 2012 [25]	36 (24 vs. 12)	62.3 vs. 61	IH	Hematoma Puncture and Aspiration in The Emergency + DC	79.2% vs. 41.6%	12
Zhang C et al., 2024 [26]	204 (102 vs. 102)	53 vs. 55	IH	Clot Removal	13.70% vs. 4.9%	3
Zhao J et al., 2012 [27]	47 (24 vs. 23)	64	mMCAI	Medical Group	12.5%–16.7% vs. 60.9%–69.6%	6–12

Note: DC = decompressive craniectomy; C = control; IH = intracerebral haemorrhage; TBI = traumatic brain injury; ASDH = acute subdural haematoma; LSCHI = large supratentorial cerebral haemorrhagic infarction; mMCAI = malignant middle cerebral artery infarction.

Overall, 1803 patients were analyzed, of whom 907 (50.3%) underwent DC. The sample sizes varied considerably, ranging from 24 participants [17] to 398 [4],[12]. Most cohorts consisted of young adult males; only three studies reported a mean age above 60 years [16],[25],[27], and three of the 14 independent cohorts included more than 50% of female participants [19],[20],[24].

### 3.2. Etiologies and comparators

The most frequently investigated etiology was TBI, examined in 6 independent cohorts [3],[4],[19],[20],[23],[24], encompassing a total of 948 patients, 474 (50%) of whom underwent DC. Other etiologies included intracerebral hemorrhage [16],[21],[25],[26], malignant middle cerebral artery infarction [27], acute subdural hematoma [18],[22], and large supratentorial cerebral hemispheric infarction [17].

In 5 studies, DC was compared with standard medical therapy [3],[4],[16],[17],[27]. The remaining trials evaluated DC against alternative surgical approaches, such as craniotomy [22], clot removal alone [26], technical variations of decompression [18]–[20],[23],[24], or DC in combination with other interventional procedures [25].

### 3.3. Follow-up duration

The follow-up duration varied substantially across the studies. Most trials reported outcomes at 6 months or earlier; a smaller number extended follow-up to 12 months [4],[15],[16],[22],[25],[27], with only one study assessing outcomes at 24 months [12].

### 3.4. Mortality

The effects of DC on mortality varied significantly among the studies (Table 2). A total of 907 patients received DC treatment, with an overall mortality rate of 24% (219 patients); the mortality rate among the 279 patients in the comparator group was 31%.

Among TBI studies, some trials reported a marked reduction in mortality in the DC group. For example, Qiu et al. [23] reported 27% versus 57%, and Hutchinson et al. [4] reported 26.9% versus 48.9% at 6 months and 30.4% versus 52% at 12 months, respectively. Conversely, other studies observed comparable or slightly elevated mortality rates in the DC arm, such as Cooper et al. [3], who reported 19% versus 18% mortality rates. Liu et al. [20] reported higher mortality in the DC group than in the comparator group receiving stepwise intracranial decompression (11.63% vs. 2.33%). Overall, mortality among TBI patients treated with DC was 32% (152 patients) compared with 41% in the control group (196 patients).

Heterogeneous findings were also observed for various etiologies. Zhao et al. [27] reported markedly lower mortality rates at 6 months (12.5% versus 60.9%) and 12 months (16.7% versus 69.6%) in patients with malignant middle cerebral artery (MCA) infarction treated with DC. Khan et al. [18] reported similar mortality rates between groups in patients with ASDH (51.16% versus 53.57%). Moussa et al. [21] reported lower mortality in the DC group for hypertensive ICH (10% vs. 25%).

These data indicated variable effects of DC on mortality across etiologies and comparators in patients with acute brain injury of either traumatic or vascular origin; however, the magnitude and direction of the mortality benefit varied across etiologies and comparators.

### 3.5. Functional outcomes

The assessment scales used to evaluate the functional outcomes were largely consistent across the studies (Table 3).

**Table 3.** Functional outcome.

Authors, Year	Outcomes	Negative Outcomes (%) Dc vs. C	Positive Outcomes (%) Dc vs. C	Range Negative Outcomes	Range Positive Outcomes	Domain ICF
Beck J et al., 2024 [16]	MRS	44%–43% vs. 58%–51%	56%–57% vs. 42%–49%	5–6	0–4	Activities & Participation (d)
Cooper DJ et al., 2011 [3]	GOS–E	70% vs. 51%	30% vs. 49%	1–4	>4	Activities & Participation (d)
Cooper DJ et al., 2020 [15]	GOS–E	59% vs. 48%	41% vs. 52%	1–4	>4	Activities & Participation (d)
Frank JI et al., 2014 [17]	MRS	71%–71% vs. 90%–70%	29%–29% vs. 10%–30%	5–6	0–4	Activities & Participation (d)
Hutchinson PJ et al., 2016 [4]	GOS–E	72.70%–68% vs. 73.4%–71.6%	27.4%–32% vs. 26.6%–28.5%	1–4	>4	Activities & Participation (d)
Khan B et al., 2016 [18]	GOS	83.87% vs. 75%	16.13% vs. 25%	1–3	4–5	Activities & Participation (d)
Kolias AG et al., 2022 [12]	GOS–E	63.7% vs. 71.2%	36.2% vs. 28.7%	1–4	>4	Activities & Participation (d)
Kumar P et al., 2023 [19]	GOS–E	66% vs. 73%	34% vs. 27%	1–4	>4	Activities & Participation (d)
Liu C et al., 2023 [20]	GOS	58.14% vs. 83. 72%	41.86% vs. 16.28%	1–3	4–5	Activities & Participation (d)
Moussa WM et al., 2017 [21]	GOS MRS	30% vs. 80%	70% vs. 20%	≥4	<4	Activities & Participation (d)
Pyne S et al., 2024 [22]	GOS–E	62.50% vs. 52.1%	37.4% vs. 47.9%	1–4	>4	Activities & Participation (d)
Qiu W et al., 2009 [23]	GOS	44% vs. 68%	57% vs. 33%	1–3	4–5	Activities & Participation (d)
Vankipuram S et al., 2020 [24]	GOS–E	49.1% vs. 46.2%	50.9% vs. 53.8%	1–4	>4	Activities & Participation (d)
Xiao B et al., 2012 [25]	BI	100% vs. 14.3%	0% vs. 85.7%	<35	≥35	Activities & Participation (d)
Zhang C et al., 2024 [26]	MRS	92.2% vs. 81.4%	7.80% vs. 18%	3–6	0–2	Activities & Participation (d)
Zhao J et al., 2012 [27]	MRS	33.3%–25% vs. 82.6%–87%	66.7%–75% vs. 17.4%–13%	5–6	0–4	Activities & Participation (d)

Note: DC = decompressive craniectomy; C = control; mRS = modified Rankin Scale; GOS = Glasgow Outcome Scale; GOS-E = extended Glasgow Outcome Scale; ICF = International Classification of Functioning, Disability and Health.

The Extended Glasgow Outcome Scale (GOS-E) was the most frequently employed, appearing in 5 of the 14 independent cohorts [3],[4],[19],[22],[24], as well as in two longer-term follow-up publications [12],[15]. The modified Rankin Scale (mRS) was used in five studies [16],[17],[21],[26],[27], the Glasgow Outcome Scale (GOS) in four studies [18],[20],[21],[23], and the BI in one study [25]. All scales fell within the International Classification of Functioning, Disability, and Health (ICF) domain of “Activities and Participation”. None of the included studies evaluated cognitive function, emotional status, social participation, or quality of life.

Outcomes were dichotomized into favorable and unfavorable categories. However, cut-off thresholds varied inconsistently across studies; for example, Zhang et al. [26] classified mRS scores of 0–2 as favorable, whereas Moussa et al. [21] used mRS scores of ≤3. These variations in dichotomization should be considered when comparing the results across trials.

Overall, functional outcomes demonstrated predominantly unfavorable results in the DC group compared with the control group across 8 of the 14 independent cohorts (Table 3). Five of these cohorts evaluated patients with TBI [20],[21],[24]. Within these studies, the percentage of TBI patients achieving a favorable outcome (GOS-E 5–8 or GOS 4–5) following DC ranged from 27.4% to 57%. Conversely, the percentage of TBI patients in the control groups who achieved a favorable outcome ranged from 26.6% to 72.1%.

When the direction of the effect was examined separately by etiology, three distinct patterns emerged (Table 4). In malignant middle cerebral artery infarction, DC reduced mortality and increased the proportion of patients with favorable functional outcomes [27], consistent with the pooled DECIMAL and DESTINY analyses [9],[28]. In diffuse TBI and deep intracerebral hemorrhage, the effect was predominantly a reduction in mortality without a parallel improvement in function, so that a larger proportion of survivors remained severely disabled. In acute subdural hematoma, the comparator was most often craniotomy rather than medical management, addressing a different clinical question (which surgical strategy is preferable) and yielding broadly comparable functional results between arms.

**Table 4.** Decompressive craniectomy effect stratified by etiology.

Etiology	Cohorts (n)	Effect on mortality	Effect on functional outcome	Predominant comparator
Malignant MCA infarction	1	Marked reduction	Improved – higher proportion of favorable outcomes	Standard medical therapy
Traumatic brain injury	6	Reduction (variable across trials)	Predominantly unchanged or worse; survivors with greater residual disability	Medical therapy or surgical variants
Intracerebral haemorrhage	4	Variable; reduction in hypertensive ICH	Mixed; frequently unfavorable in deep ICH	Medical therapy, clot removal, or hematoma evacuation
Acute subdural haematoma	2	Comparable between arms	Comparable; no clear DC advantage	Craniotomy (distinct clinical question)
Large supratentorial infarction	1	Comparable between arms	Limited, confounded data	Combined surgical intervention

Note: DC = decompressive craniectomy; MCA = middle cerebral artery; TBI = traumatic brain injury; ICH = intracerebral haemorrhage. The directions of effect were descriptive and based on group-level data from the included trials.

Notable exceptions were observed in two of these studies. Moussa et al. [21], comparing DC with hematoma evacuation alone in hypertensive ICH, reported favorable outcomes in 70% of DC patients versus 20% of controls, assessed using the mRS at 6 months. Zhao et al. [27], comparing DC with standard medical therapy in patients with malignant MCA infarction, reported favorable outcomes in 66.7% of patients receiving DC versus 13% of controls at 6 months.

The publication by Xiao et al. [25] warrants a separate discussion. Here, patients with large supratentorial intracerebral hemorrhage were enrolled, revealing that none of the survivors in the DC group achieved a favorable outcome on the BI (BI ≥ 35) at 12 months, in contrast to 85.7% of survivors in the control group (emergency puncture/aspiration followed by craniectomy). However, the study design differed from others, as both arms received craniectomy, with the experimental variable being the addition of prior emergency hematoma evacuation. Consequently, the control group received a more intensive combined intervention, potentially explaining the superior functional outcomes observed in this group.

### 3.6. Rehabilitation data

Analysis of the extracted data revealed a notable lack of information concerning postoperative rehabilitation (Table 5). Only one study [22] explicitly reported the number of patients admitted to an inpatient rehabilitation program (36 vs. 45); however, it did not describe the treatment intensity, duration, timing, or specific modalities. All other studies failed to describe any rehabilitation protocol or specify whether rehabilitation occurred in inpatient, outpatient, or home-based settings.

**Table 5.** Postoperative rehabilitation.

Authors, Year	Rehabilitation (Y vs. N)	Rehabilitation Setting (Inpatient/Outpatient/Home)	Frequency	Intensity	Type	Time
Beck J et al., 2024 [16]	nd	nd	nd	nd	nd	nd
Cooper DJ et al., 2011 [3]	nd	nd	nd	nd	nd	nd
Cooper DJ et al., 2020 [15]	nd	nd	nd	nd	nd	nd
Frank JI et al., 2014 [17]	nd	nd	nd	nd	nd	nd
Hutchinson PJ et al., 2016 [4]	nd	nd	nd	nd	nd	nd
Khan B et al., 2016 [18]	nd	nd	nd	nd	nd	nd
Kolias AG et al., 2022 [12]	nd	nd	nd	nd	nd	nd
Kumar P et al., 2023 [19]	nd	nd	nd	nd	nd	nd
Liu et al., 2023 [20]	nd	nd	nd	nd	nd	nd
Moussa W et al., 2017 [21]	nd	nd	nd	nd	nd	nd
Pyne S et al., 2024 [22]	36 vs. 45	inpatient	nd	nd	nd	nd
Qiu W et al., 2009 [23]	nd	nd	nd	nd	nd	nd
Vankipuram S et al., 2020 [24]	nd	nd	nd	nd	nd	nd
Xiao B et al., 2012 [25]	nd	nd	nd	nd	nd	nd
Zhang C et al., 2024 [26]	nd	nd	nd	nd	nd	nd
Zhao J et al., 2012 [27]	nd	nd	nd	nd	nd	nd

Note: Y = yes; N = no; nd = not described.

## 4. Discussion

In this systematic review of 16 RCTs, we examined the long-term functional outcomes following DC in patients with severe brain injuries, utilizing data from 14 independent populations and 1803 patients.

### 4.1. Survival versus functional recovery

Our primary finding was a disconnect between the mortality advantage of DC compared to control populations and the lack of a corresponding advantage in functional recovery in the DC group. Across the studies analyzed, DC appeared to reduce mortality, particularly among patients with TBI, who comprised approximately 53% of the total population, and in patients with other etiologies. Conversely, in 8 of the 14 independent cohort analyses, a greater proportion of patients in the DC group exhibited less-than-optimal functional recovery at follow-up than the control group. Specifically, within TBI studies, only 3 of 6 cohorts demonstrated a higher rate of favorable outcomes in the DC arm, suggesting that increased survival does not necessarily translate into improved global functioning or social participation. These findings align with previous non-randomized evidence and likely reflect the fact that DC, by reducing mortality, increases the proportion of survivors with severe residual disabilities [5],[29]. Evidence from cohorts of patients with severe acquired brain injury further supports the importance of baseline clinical characteristics and age as key prognostic factors influencing discharge outcomes and functional trajectories [30]. These elements should be considered when interpreting the long-term results of decompressive craniectomy.

However, this overall pattern conceals important differences between pathologies, and the results are therefore best interpreted by etiology. In malignant MCA infarction, DC consistently improves survival and functional outcomes, and the disability–survival paradox does not apply in the same way; the benefit is unequivocal in appropriately selected patients. In diffuse TBI and deep ICH, by contrast, the advantage is largely confined to survival, and the additional survivors are concentrated in the more severely disabled outcome categories. In ASDH, the comparison is typically against craniotomy; therefore, the relevant question is the choice of surgical technique rather than surgery versus medical care. Age is a powerful effect modifier of this balance: In the three cohorts with a mean age above 60 years, the same mortality reduction is more likely to yield survivors with severe disability because older patients have a lower neuroplastic reserve and a higher burden of comorbidity. The disability–survival paradox is thus most pronounced in older patients and in non-stroke etiologies, and age should be weighed explicitly in prognostication and shared decision-making.

These results are consistent with the “disability-survival paradox”, a phenomenon wherein increased survival in studies of severe brain injury is associated with elevated levels of disability [31]. When DC prevents deaths that would otherwise occur, it generates a new category of “survivors with severe disability” rather than solely decreasing the number in the “dead” category [32]. Consequently, DC does not worsen individual outcomes but rather expands the survivor population to include individuals with significantly more severe residual impairment. From a clinical perspective, this necessitates informed consent discussions that explain the risk of surviving with substantial disability and reinforce the need for postoperative rehabilitation plans; however, this factor is infrequently documented in trial research.

Notably, mortality rates for patients appear to be stable after 12 months, as observed in the RESCUEicp study, which showed minimal improvement in mortality rates between 6 and 12 months (26.9% vs. 30.4%) in the DC group [4],[15], indicating that the survival advantage of DC is primarily evident within the first 12 months post-surgery. The implications of this finding are significant: If the mortality rate is stabilized over time but the long-term potential for functional recovery continues to evolve, extended follow-up will be needed to fully appreciate the relationship between mortality and disability.

Finally, the evaluation of functional outcomes was complicated by the heterogeneity of the comparison groups. Five studies compared DC with standard medical management, whereas the remaining 9 studies compared DC with alternative forms of surgical intervention. These groups address distinct clinical questions; the first set evaluates whether cranial vault surgical decompression provides an advantage, whereas the second set assesses which form of surgical intervention yields better outcomes in terms of recovery. Consequently, the type and extent of surgical intervention may affect the timing of rehabilitation initiation and the rehabilitation potential of the survivors.

### 4.2. Limitations of outcome assessment

These results may have been influenced by several factors, including the timing of DC, premorbid clinical status, patient comorbidities, variability in follow-up duration, and differences in the early post-injury rehabilitation pathways.

Several surgical and procedural factors, although not directly evaluated in the included trials, are likely to modulate these outcomes and should be considered in the design of future studies [4]. The size and laterality of the bone flap should be individualized, and the most common techniques in adults involve unilateral fronto-temporal-parietal craniectomy [33] and bifrontal craniectomy [34]. The timing of decompression also influences the degree of pressure relief and potential secondary injury, with earlier decompression generally favored to limit damage from uncontrolled intracranial hypertension, as earlier interventions may offer greater neuroprotective benefits [35]; therefore, DC should be performed as soon as possible after the injury [36],[37]. In addition, the optimal timing of cranioplasty, the replacement of the bone flap or an artificial substitute to restore normal cranial anatomy [38], and the well-recognized complications of DC (including infection, wound inflammation, cerebral hemorrhage, hydrocephalus, cerebrospinal fluid fistula, subdural hygroma, and the syndrome of the trephined) can shape the rehabilitation trajectory and the functional ceiling that survivors can reach [39]. Because these variables are inconsistently reported, their contribution to long-term outcomes cannot be quantified from this evidence and represents a priority for prospective data collection.

All functional scales of the outcome assessments utilized across the reviewed studies–the GOS-E, mRS, GOS, and BI–primarily classified patients under “Activities and Participation” within the ICF framework. These scales measure patient disability by placing individuals into broad categories rather than providing a detailed view of specific functional limitations, remaining capabilities, or an individual's ability to adapt. Moreover, none of the reviewed studies employed multidimensional assessments that evaluated aspects of patient cognition, emotional well-being, social reintegration, or quality of life. The use of broad functional outcome assessments can lead to a gross underestimation of the impact of a patient's residual disability following a DC and does not enable researchers to fully understand the complexities of a patient's recovery process after a DC. Therefore, future clinical trials examining the effects of treatment options following a DC event should consider employing tools that assess multiple areas of the ICF framework, such as standardized cognitive batteries, measures of participation and community integration, and patient-reported outcome measures evaluating quality of life.

In addition to the aforementioned limitations, the way in which outcomes were grouped into favorable and unfavorable groups, although required to facilitate cross-trial comparisons, was done using different threshold values across trials (for example, mRS 0–2, 0–3, and 0–4 were each considered “favorable”), which limits the degree to which aggregated data across trials can be compared.

### 4.3. The missing rehabilitation dimension

The analysis of rehabilitation data revealed an interesting finding in this review. Only Pyne et al. [22] in their independent cohort, examined the rehabilitation data. They provided limited information on inpatient rehabilitation. However, they did not describe the length, intensity, or methods of rehabilitation. Conversely, none of the remaining studies measured or reported the rehabilitation. This should be interpreted as rehabilitation being unmeasured rather than necessarily absent. Several of the included trials were conducted in healthcare systems (e.g., the United Kingdom and Australia) in which structured inpatient neurorehabilitation is a routine standard of care; therefore, it is likely that most survivors did receive rehabilitation that was not captured in the trial datasets. Therefore, the problem is one of trial reporting rather than patients being left without rehabilitation.

The absence of rehabilitation data suggests two key limitations in understanding rehabilitation concerning DC. First, we could not assess the extent to which variations in rehabilitation pathways contributed to the observed variability in functional outcomes across the trials. Second, we do not know whether DC is a neutral or negative prognostic factor for rehabilitation outcomes, a critical question given the complexity of the recovery process following craniectomy, which may involve complications such as hydrocephalus, subdural hygroma, syndrome of the trephined, and increased operative risk of subsequent cranioplasty [39].

A substantial body of evidence describes the disability-survival paradox, in which the observation that DC produces a greater number of survivors with high levels of disability highlights the critical role of postoperative rehabilitation quality and quantity in determining long-term disability severity. Consequently, without comprehensive rehabilitation data collection and reporting, the literature cannot adequately examine the most clinically relevant question: Not whether DC saves lives but whether survivors can achieve significant functional recovery [31].

Therefore, future RCTs should collect prospective rehabilitation data, including the site of care (inpatient, outpatient, or home-based), date rehabilitation began, intensity and frequency of rehabilitation, and types of interventions used. Structured long-term care models have been proposed for other severe neurological conditions, such as multiple sclerosis, where home-based palliative approaches have been systematically evaluated to address complex disabilities and caregiver burden [40]. A similar multidimensional framework could be considered for patients surviving DC, particularly those with severe residual disability. Collecting these data would enable researchers to determine the relative roles of surgery and rehabilitation in long-term functional recovery.

### 4.4. Limitations

This systematic review had several limitations. First, and most importantly, the search was restricted to PubMed and Scopus and did not include Embase or the Cochrane Central Register of Controlled Trials (CENTRAL). For an intervention investigated in several large international trials, the omission of these databases is a significant methodological limitation that may have led to the non-retrieval of eligible RCTs from our analysis. The completeness of the evidence base presented here should be interpreted with these constraints in mind. Future updates of this review should incorporate Embase and CENTRAL with trial registries to ensure comprehensive case ascertainment. Second, considerable clinical and methodological diversity among the trials, including variations in etiology, surgical technique, comparison groups, outcome measurement scales, dichotomization thresholds, and follow-up duration, prevented a quantitative meta-analysis and further limited the ability to make cross-study comparisons. Third, most studies reported outcomes at 6 months or earlier, which may be insufficient to fully capture the extent of neurological recovery following severe TBI, given that TBI-related impairments may persist and evolve over years. Finally, a formal assessment of the certainty of evidence, such as using the GRADE framework, was not conducted. This limitation is particularly relevant for disability and quality-of-life outcomes, which may require follow-up periods of 24 months or longer to capture the full trajectory of recovery and adaptation, as these dimensions evolve more slowly than mortality.

A risk bias assessment using the RoB 2 tool indicated that most included studies (11 of 16) were judged to have “some concerns”. Three studies were rated as high risk [18],[19],[26] and two were rated as low risk [15],[16]. All 16 studies received a judgment of “some concerns” for Domain 2 (deviations from intended interventions), reflecting the inherent impossibility of blinding in surgical trials rather than a methodological flaw. The three high-risk studies were primarily downgraded due to unblinded outcome assessment [18],[26] or incomplete handling of missing outcome data, particularly in the context of high mortality [19]. Consequently, the results of these studies should be interpreted with caution. While several larger multicenter studies (DECRA, RESCUEicp, SWITCH, RESCUE-ASDH) were well-designed, some smaller single-center studies lacked trial registries, employed unblinded outcome assessors, or did not explicitly state an intention-to-treat analysis, which limited confidence in the overall evidence base.

## 5. Conclusions

Decompressive craniectomy remains an effective, life-saving intervention for refractory intracranial hypertension, and its benefits on functional outcomes are clearest in malignant MCA infarction. The results of this systematic review indicate a paucity of studies examining the effects of DC from a rehabilitative and functional perspective over an extended period. Moreover, rehabilitation pathways for these patients are not standardized. Well-designed prospective studies evaluating neurological outcomes and individual rehabilitation pathways are required to determine the true impact of DC on functional outcomes and support efforts toward standardizing the rehabilitation process.

This lack of information prevents the establishment of meaningful correlations between rehabilitation pathways and observed functional outcomes, making it difficult to distinguish the contribution of rehabilitation from the natural course of neurological recovery in the brain-injured population. Additionally, it is difficult to determine, based on the data available in the literature, whether DC is a negative prognostic factor for achieving rehabilitation goals after severe acquired brain injury and whether DC may affect intensive rehabilitation outcomes.

## Use of AI tools declaration

The authors declare they have not used Artificial Intelligence (AI) tools in the creation of this article.
